# Fungal Root Microbiome from Healthy and Brittle Leaf Diseased Date Palm Trees (*Phoenix dactylifera* L.) Reveals a Hidden Untapped Arsenal of Antibacterial and Broad Spectrum Antifungal Secondary Metabolites

**DOI:** 10.3389/fmicb.2017.00307

**Published:** 2017-02-28

**Authors:** Fedia B. Mefteh, Amal Daoud, Ali Chenari Bouket, Faizah N. Alenezi, Lenka Luptakova, Mostafa E. Rateb, Adel Kadri, Neji Gharsallah, Lassaad Belbahri

**Affiliations:** ^1^Laboratory of Plant Biotechnology, Faculty of Science, University of SfaxSfax, Tunisia; ^2^Biotechnology, NextBiotechAgareb, Tunisia; ^3^Graduate School of Life and Environmental Sciences, Osaka Prefecture UniversitySakai, Japan; ^4^Department of Biology and Genetics, Institute of Biology, Zoology and Radiobiology, University of Veterinary Medicine and PharmacyKosice, Slovakia; ^5^School of Science and Sport, University of the West of ScotlandPaisley, UK; ^6^College of Science and Arts in Baljurashi, Al Baha UniversityAl Bahah, Saudi Arabia; ^7^Laboratory of Soil Biology, University of NeuchâtelNeuchâtel, Switzerland

**Keywords:** endophytic fungi, secondary metabolites, brittle leaf disease, antimicrobial activity, date palm, enzymes

## Abstract

In this study, we aimed to explore and compare the composition, metabolic diversity and antimicrobial potential of endophytic fungi colonizing internal tissues of healthy and brittle leaf diseased (BLD) date palm trees (*Phoenix dactylifera* L.) widely cultivated in arid zones of Tunisia. A total of 52 endophytic fungi were isolated from healthy and BLD roots of date palm trees, identified based on internal transcribed spacer-rDNA sequence analysis and shown to represent 13 species belonging to five genera. About 36.8% of isolates were shared between healthy and diseased root fungal microbiomes, whereas 18.4 and 44.7% of isolates were specific to healthy and BLD root fungal microbiomes, respectively. All isolates were able to produce at least two of the screened enzymes including amylase, cellulase, chitinase, pectinase, protease, laccase and lipase. A preliminary screening of the isolates using disk diffusion method for antibacterial activity against four Gram-positive and three Gram-negative bacteria and antifungal activities against three phytopathogenic fungi indicated that healthy and BLD root fungal microbiomes displayed interesting bioactivities against examined bacteria and broad spectrum bioactivity against fungal pathogens. Some of these endophytic fungi (17 isolates) were fermented and their extracts were evaluated for antimicrobial potential against bacterial and fungal isolates. Results revealed that fungal extracts exhibited antibacterial activities and were responsible for approximately half of antifungal activities against living fungi. These results suggest a strong link between fungal bioactivities and their secondary metabolite arsenal. EtOAc extracts of *Geotrichum candidum* and *Thielaviopsis punctulata* originating from BLD microbiome gave best results against *Micrococcus luteus* and *Bacillus subtilis* with minimum inhibitory concentration (MIC, 0.78 mg/mL) and minimum bactericidal concentration (6.25 mg/mL). *G. candidum* gave the best result against *Rhizoctonia solani* with MIC 0.78 mg/mL and minimum fungicidal concentration (MFC, 6.25 mg/mL). In conclusion, using plant microbiomes subjected to biotic stresses offers new endophytes with different bioactivities than those of healthy plants. Therefore, date palm endophytic fungi represent a hidden untapped arsenal of antibacterial and broad spectrum antifungal secondary metabolites and could be considered promising source of bioactive compounds with industrial and pharmaceutical applications.

## Introduction

The recent development of antimicrobial resistance by pathogenic microorganisms intimidates the current treatment and prevention of ever-increasing range of infections by bacteria and fungi and leads to new microorganisms that cannot be controlled by the drugs that referred to as “superbugs” (Vargiu et al., 2016). Reports on superbugs such as MRSA (methicillin-resistant *Staphylococcus aureus*) and NDM-1 (New Delhi metallo-beta-lactamase 1) strains are rising terrifyingly nowadays and represent a serious threat to public health (Khan and Khan, 2016). The most important causes that favored antibiotic resistance include, excessive and inappropriate antibiotic use among humans and animals (Kaye, 2016), environmental contamination with antibiotics (Lammie and Hughes, 2016), poor hygienic conditions (Senn et al., 2016), global trade and travel (Riddle and Connor, 2016), medical tourism (Saliba et al., 2016), and a decline in new antibiotic development (Marston et al., 2016).

Therefore, there is an increasing demand for new bioactive, cost-effective and sustainable antimicrobial molecules in medicine, industry and agriculture that prompted the development of diverse programs such as screening for new plants or fungal species with antimicrobial activities from unexplored ecological niches and habitats (Lugtenberg et al., 2016). Historically, plants were considered potential source of drugs for treatment of many human diseases. Certain endophytes could also produce the same or similar compounds as their host plants (Xu et al., 2009). Since the discovery of the billion-dollar anticancer drug Taxol from the endophytic fungus of *Taxus brevifolia* namely *Taxomyces andreanae*, endophytes have gained increasing interest of mycologists, botanists, pharmacologists, and even plant pathologists (Stierle et al., 1993; Venugopalan and Srivastava, 2015; Gouda et al., 2016; Newman and Cragg, 2016). Recently, considerable knowledge has accumulated about endophytes, defined as a community of microorganisms that colonize all plant species tissues without causing any apparent symptoms (Petrini et al., 1992). Endophytic microorganisms offer many ecological and physiological benefits to their host plants including adaptability to different kinds of stress, growth promotion and resistance against plant pests and pathogens (Jia et al., 2016).

Therefore, several attempts have been made to study the biology of endophytic microorganisms and to exploit the untapped potential of their bioactive compounds (Mousa and Raizada, 2013). Consequently, numerous endophytes including bacteria, fungi, and actinomycetes had been recovered and characterized from approximately 300,000 plant species (Strobel and Daisy, 2003). Accordingly, recent studies have shown that endophytic fungi represent a promising resource of novel natural products with biological importance such as antidiabetic, anticancer, antioxidant, antimicrobial, antiviral, and immunosuppressive activities (Katoch et al., 2015; Chen et al., 2016; Monggoot et al., 2016; Rathna et al., 2016). These bioactive compounds may be classified as alkaloids, steroids, terpenoids, flavonoids, benzopyranones, quinones, isocumarins, phenolics, tetralones, lactones, peptides, and many other subclasses (Xu et al., 2008; Kaul et al., 2012). The discovery of novel antimicrobial compounds from endophytic fungi constitutes an important alternative to overcome numerous recent problems such as the insufficiency of current antibiotics against human pathogens, the wider range of infections and the low rate of new antimicrobial agent discovery (Strobel et al., 2001; Marston et al., 2016). Antimicrobial compounds could also be used as food preservatives among other biotechnological applications (Haque et al., 2015).

Different environmental factors in addition to biotic and abiotic stresses are considered the main drivers of plant fungal endophyte diversity and believed to shape their communities. Although endophyte characterization in healthy tissues have been extensively studied, few reports addressed such communities in plants under abiotic or biotic stresses (Douanla-Meli et al., 2013). Additionally, Date palm trees, extensively used in folk medicine, have been shown to be rich with numerous secondary metabolites with interesting pharmacological and antimicrobial activities (Abdennabi et al., 2016). Therefore, endophytes of this plant species were the main focus of this study, with a double objective. First, endophytes from healthy and brittle leaf diseased (BLD) root tissues have been recovered and identified. Second, endophyte communities have been compared in terms of metabolic potential by testing enzyme activities of amylase, cellulase, chitinase, pectinase, protease, laccase and lipase and antimicrobial bioactivities against four Gram-positive and three Gram-negative bacterial species and antifungal activities against three phytopathogenic fungi. Contribution of secondary metabolites to the bioactivities of endophytes is then screened using their EtOAc culture extracts.

## Materials and Methods

### Isolation of Root Fungal Endophytes from Healthy and BLD Date Palm Trees

Roots of healthy and BLD adult date palm trees variety Deglet Ennour were collected from groves located in Nefta Oasis, near to Algerian-Tunisian border and just at the north of Chott Djerid (Latitude: 33° 52′ 23.12″ N, Longitude: 7° 52′ 39.54″ E). Numerous samples from roots and leaves were collected (*n* = 20). Plant materials were transferred to the laboratory in sterile bags and stored at 4°C until processing. Endophytic fungi were isolated from the internal tissues of date palm roots, as described by Hallmann et al. (2007). Briefly, samples were washed with tap water for 30 min and surface sterilized by sequential washes in 70% ethanol followed by 3% NaClO for 3 min. Finally, the sterile plant material was washed several times with sterilized distilled water and cut into small fragments (0.5–1 cm) under sterile conditions using a sterile scalpel. In total, 103 root fragments were used for fungal isolation. Surface sterilized plant material was placed on potato dextrose agar (PDA) media supplemented with 100 μg/mL streptomycin for bacterial growth inhibition. The plates were incubated for 3–5 days at 30°C until fungal mycelia were observed. Single fungal isolates have been obtained by sub-culturing emergent hyphal tips. Cultures were preserved on PDA slants for subsequent morphological and molecular identification and biochemical characterization.

### Fungal DNA Extraction and Amplification

Pure cultures of the isolates on PDA slant vials were selected for DNA extraction. Mycelia were excised from 5 days old plates. The extraction was processed using the DNA-Easy Plant Mini kit (QIAGEN, Basel, Switzerland) following manufacturer’s protocol. The quantity and quality of the genomic DNA were evaluated by a NanoDrop NT-100 UV spectrophotometer (Witec AG, Switzerland) and by visual observation through 1.5% agarose gel electrophoresis. The molecular identification of the endophytic fungi isolated from the roots of healthy and BLD date palm trees was carried out as described by Paul et al. (2008). The internal transcribed spacer (ITS) of rDNA was amplified by the polymerase chain reaction using primers, ITS1 (TCCGTAGGTGAACCTGCGG) and ITS4 (TCCTCCGCTTATTGATATGC) (White et al., 1990). All reactions were carried out in a total volume of 50 μL, containing 5 μL 10x Ex Taq buffer (20 mM Tris–HCl, pH 8.0, 100 mM KCl), 4 μL 2.5 mM dNTP mixture, 0.5 μM of each primer, 1.25 units Taq DNA polymerase (Takara Bio, Ohtsu, Japan) and 10 ng DNA. The amplifications were performed using a master gradient thermal cycler (Eppendorf, Basel, Switzerland) with the following cycling profile: Denaturation step at 95°C for 2 min followed by 30 cycles including denaturation at 94°C for 20 s, annealing at 55°C for 25 s and extension at 72°C for 15 s and final extension step at 72°C for 10 min. PCR amplicons were purified with Minelute PCR purification kit (Qiagen, Basel, Switzerland) according to the manufacturer’s specifications (Belbahri et al., 2006).

### DNA Sequencing and Phylogenetic Analysis

The purified amplicons were sequenced in both directions using the same PCR primers and BigDye^®^ Terminator v. 3.1 cycle sequencing kit. Sequencing reactions were resolved on an ABI 3130 XL available at the iGE3 [Institute of Genetics and Genomics in Geneva, University of Geneva Medical Center (CMU), Switzerland]. Raw sequence files were manually edited using SeqMan^TM^II (DNASTAR, Madison, WI, USA) and a consensus sequence was generated for each sequence. The consensus sequence for genomic region was blasted against the NCBI’s GenBank sequence database using megablast to identify their closest species relatives. For the gene region, the retrieved sequences from GenBank together with sequences generated in this study, were aligned using the multiple sequence alignment web-based MAFFT program (Katoh and Toh, 2008). Phylogenetic trees were constructed based on the maximum-likelihood (ML) algorithm (Felsenstein, 1981) using MEGA6 (Tamura et al., 2013), with evolutionary distances computed using the Kimura 2-parameter model (Kimura, 1980). Validity of branches in the resulting trees was evaluated by bootstrap resampling support of the data sets with 1000 replications.

### Screening for Extracellular Enzymes

#### Screening for Amylase Activity

Amylase-producing fungi were characterized using glucose-yeast-peptone (GYP) medium containing (g/L): glucose (1), yeast extract (0.5), peptone (0.5), agar (15), and supplemented with 0.2% of starch. The pH of the medium was adjusted to 6.0. Mycelia of each endophytic fungus were placed in the center of the plates and incubated at 30°C for 72 h. After incubation, an iodine solution (*I*_2_ = 1 g, KI = 2 g/300 ml) was poured on the plates. The appearance of clear zones around the colonies indicated the presence of amylase activity (Saleem and Ebrahim, 2014).

#### Screening for Cellulolytic Activity

Cellulolytic activity from endophytic fungi was characterized by inoculating GYP agar supplemented with 0.5% carboxymethyl cellulose (CMC). After incubation for 72 h at 30°C, the plates were overlaid with 1% red congo and distained with 1 M NaCl. Strains with clear zones around the colonies were mentioned as cellulolytic enzyme producers (Shahriarinour et al., 2011).

#### Screening for Chitinase Activity

A minimum medium containing (g/L): colloidal chitin (10 g), (NH4)_2_SO_4_ (2 g), KH_2_PO_4_ (0.7 g), HgSO_4_.7 H_2_O (0.5 g), FeSO_4_. 7H_2_O (0.01 g), agar (15 g) was used for chitinase screening (Jenifer et al., 2014). Colloidal chitin was prepared following the protocol described by Nagpure and Gupta (2012).

#### Screening for Pectinase Activity

Pectinolytic activity of recovered endophytic fungi were evaluated on the medium based on the method described by Nitinkumar and Bhushan (2010). After incubation of inoculated plates for 72 h at 30°C, cetrimonium bromide (CTAB) solution (1%) was poured onto colonies for detection of pectinase producing fungi.

#### Screening for Laccase Activity

The fungal isolates were inoculated on malt extract medium with the following composition (g/L): malt extract (30), agar (20) supplemented with 2 mM ABTS (2,2′-azino-di-3-ethylbenzotiazol-6-sulfonate acid) and incubated at 30°C. Positive laccase activity was recorded by the appearance of reddish brown halo around the inoculated strains indicating the presence of laccase activity (Gnanasalomi and Gnanadoss, 2013).

#### Screening for Lipase Activity

Lipase activity was assessed using the protocol described by Thota et al. (2012). The endophytic fungi were inoculated on medium containing (g/L): 8 nutrient broth, 4 sodium chloride, 10 agar supplemented after autoclaving with olive oil (16.34 mL), Tween 80 (250 μL) and Rhodamine solution (10 μg/mL). After incubation at 30°C, lipase activity was detected by irradiating the plates with Ultra Violet light at 350 nm.

#### Screening for Protease Activity

Protease activity was checked using a simple medium containing (g/L): yeast extract (3), casein peptone (5), agar (15) supplemented after autoclave with 250 mL of sterile skimmed milk (Mohanasrinivasan et al., 2012). The fungal isolates were inoculated on the center of plates. After incubation for 72 h at 30°C, the appearance of clear zones around them indicated the ability of the isolates to produce and secrete protease in the medium.

### Screening for Secondary Metabolites Production

#### Assay of Microorganisms

Among microorganisms used for antimicrobial assay, seven were bacteria including four Gram-positive bacteria (*Bacillus cereus* ‘Bc’ JN 934390, *B. subtilis* ‘Bs’ JN 934392, *Staphylococcus aureus* ‘Sa’ ATCC 6538 and *Micrococcus luteus* ‘Ml’) and three Gram-negative bacteria (*Salmonella enteritidis* ‘Se’ ATCC 43972, *Escherichia coli* ‘Ec’ ATCC 25922, and *Klebsiella pneumonia* ‘Kp’). Bacterial cultures were prepared in 10 mL of Mueller-Hinton broth (MHB) (Mueller and Hinton, 1941) and maintained at 30°C with constant shaking (180 rpm). For the studied bacteria, the optical density at 600 nm of overnight cultures was adjusted to 0.1 corresponding to 3.2 × 10^6^ CFU/mL.

Antifungal activity was conducted using three phytopathogenic fungi including *Rhizoctonia solani* ‘Rs,’ *Fusarium oxysporum* ‘Fo’ AB586994 and *Pythium catenulatum* ‘Pc’ AY598675. The examined fungi were inoculated on PDA plates and incubated for 7 days at 30°C.

### *In vitro* Antimicrobial (Antagonistic) Assay

The endophytic fungi were subjected to antimicrobial assays that allows rapid but qualitative selection of the bioactive isolates. For antibacterial activity, the disk diffusion method was carried out as described by Ezra et al. (2004). Briefly, plugs from 5-days-old pure culture of each endophytic fungi (6 mm diameter) were cut using sterile Pasteur pipette and placed onto the periphery of Mueller-Hinton agar (MHA) plates initially inoculated with 100 μL of culture of the tested bacteria. Diffusion was carried out for 2 h at 4°C. After incubation of plates for 24 h at 30°C, the antibacterial activity was expressed by measuring the diameters of inhibition zones around the fungal plugs.

The antifungal activity of the isolated fungal endophytes was assessed by dual culture method according to Alenezi et al. (2016). A dual culture was conducted on PDA plates with plugs from pure culture (10 mm in diameter) from the two partners, endophytic fungus and tested phytopathogenic fungi. The control plates were inoculated only with the phytopathogenic fungi. The plates were incubated for 72 h at 30°C and the percentage of inhibition (IP) of each isolate was recorded. All experiments were conducted in triplicates. The percentage of inhibition was calculated as the following formula (A: radial diameter of phytopathogenic fungus in control plate, B: radial diameter of phytopathogenic fungus in dual culture plate)

IP=[(A-B)÷A]×100

### Secondary Screening: Fermentation in Liquid Medium and Antimicrobial Activity

The endophytic isolates from healthy and BLD date palm roots that exhibited inhibitory activity against the largest number of examined bacteria and fungi were selected for secondary screening of bioactive metabolites production (Santos et al., 2015). Endophytic fungi were cultured on PDA plates for 5 days at 30°C. Then, three plugs (5 mm × 5 mm) with fungal culture were transferred to 300 mL flasks containing 150 mL of autoclaved potato dextrose broth (PDB). The cultures were incubated in a shaker at 30°C, 150 rpm for 21 days until stationary phase reached. Afterward, the fungal mycelia were separated from culture by filtration. The resulting filtrates from each endophytic fungus were extracted with equal volume of EtOAc. The organic solvent was evaporated under reduced pressure using rotary evaporator. The fungal extracts were then dissolved in DMSO or double distilled water to obtain final concentration of 100 μg/μL. Finally, the concentrated fungal extracts were passed through 0.2 μm filtration membrane and assayed for their antimicrobial activity by agar diffusion method. Briefly, 100 μL of freshly prepared culture for bacteria or spore suspension (10^6^ spores/mL) for fungi were seeded onto agar plates surface using a sterile swab cotton. Each well was then filled with 60 μL of endophytic fungi extracts. The plates were kept for 2 h at 4°C to facilitate the diffusion of fungal filtrate. Afterward, they were incubated at 37°C for 24 h for bacteria and at 30°C for 3–5 days for fungal strains. Antimicrobial activity was evaluated by measuring the diameter of inhibition zones around the wells. All experiments were carried out in duplicate.

### Determination of Minimum Inhibitory Concentration (MIC), Minimum Bactericidal Concentration (MBC), and Minimum Fungicidal Concentration (MFC) of Endophytic Fungal Extracts

The minimum bactericidal concentration (MBC) and minimum fungicidal concentration (MFC) were determined using broth micro-dilution method in a sterile 96 well micro plate, as described by Gulluce et al. (2007). Serial dilution of each fungal extract was prepared to get final concentrations ranging from 0.781 to 100 μg/μL. Each well was supplemented with 10 μL of bacterial or fungal suspension, 90 μL of liquid culture broth and 100 μL of fungal extract. The last well containing the above-cited components without addition of the fungal extract was considered as positive control. The one containing DMSO without extract was the negative control. Plates were incubated at 37°C for 24 h for bacterial strains and for 3 days at 30°C for fungal strains. Afterward, 25 μL of MTT (3-(4,5-dimethylthiazol-2-yl)-2,5-diphenyltetrazolium bromide] was added to each well for evaluation of the microorganisms viability. After incubation of plates for 30 min at 37°C, the clear wells indicated the inhibition of cell growth. The MIC is defined as the lowest concentration of extract that inhibit the growth of microorganisms. The MBC values were determined after incubation of plates for 48 h at 37°C as the highest dilution of extract that completely inhibit the growth of bacteria. MFC was considered as the first well with no visible growth of the test fungi. It was determined by inoculating the PDA plates with 10 μL of the well content followed by incubation for 3–5 days at 30°C. The MFC values were interpreted as the lowest concentration of endophytic fungal extract that inhibits fungal growth.

### Statistical Analysis

Data were analyzed using IBM SPSS statistics by one-way analysis of variance (ANOVA) and independent-samples *T*-test. The level of significance used for all statistical tests is 5% (*p* < 0.05).

## Results

### Phylogenetic Affinities of Endophytic Fungi

**FIGURE 1:**
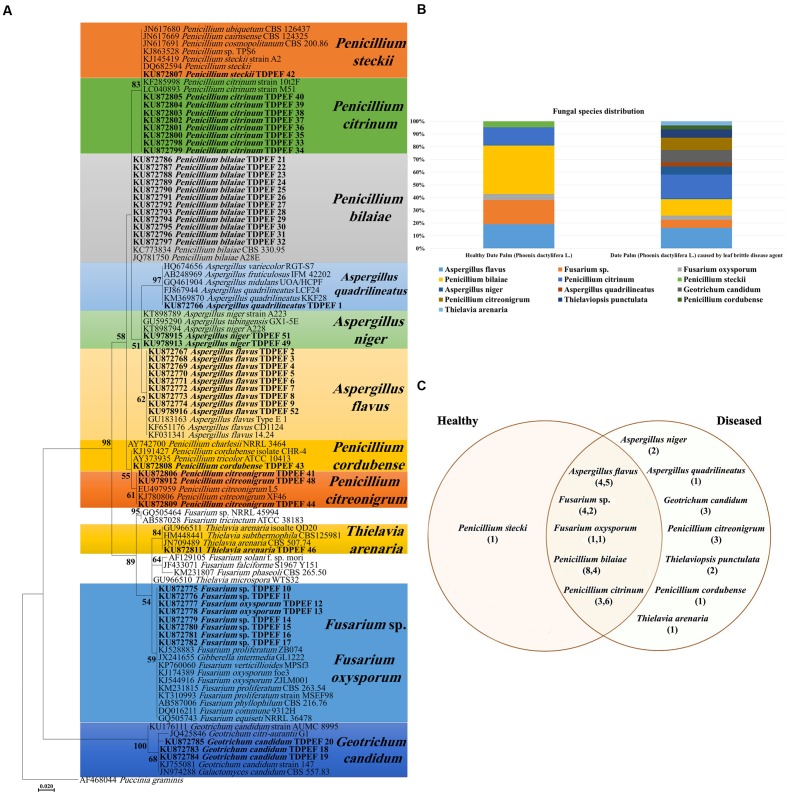
**(A)** Maximum Likelihood phylogenetic tree of fungal endophytes recovered from healthy and brittle leaf diseased (BLD) diseased date palm roots. **(B)** Fungal species composition in healthy and BLD roots. **(C)** Venn diagram showing the distribution of endophytic isolates in plants roots (the first number between brackets represents the number of isolates recovered from healthy roots and the second number represents that recovered from roots of LBD roots).

Endophytic fungi ITS-rDNA sequence analysis revealed diverse taxonomic affinities among the isolates (**Figures 1A,B**). In total, 52 ascomycetes were obtained in the present study. About 21 of them were isolated from healthy date palm roots, the remaining isolates were recovered from BLD roots (**Figures 1A,B**). Isolates belonged to Eurotiomycetes (*n* = 38), Sordariomycetes (*n* = 11) and Saccharomycetes (*n* = 3). Isolates comprised six genera with highest abundance of *Penicillium* (26 isolates) and *Aspergillus* (12 isolates). Among *Penicillium* species isolated, 12 isolates were identified as *P. bilaiae*, nine as *P. citrinum*, three as *P. citreonigrum*, one as *P. steckii*, and one as *P. cordubense*. The 12 *Aspergillus* species comprised one isolate of *A. quadrilineatus*, nine as *A. flavus*, and two as *A. niger*. Eight *Fusarium* were isolated from roots of date palm including six *Fusarium* sp. and two *F. oxysporum*. In Addition, two, three and one isolate of *Thielaviopsis punctulata, G. candidum and Thielavia arenaria*, respectively, were isolated only from BLD date palm roots (**Figures 1B,C**).

### Screening for Enzyme Production

**FIGURE 2:**
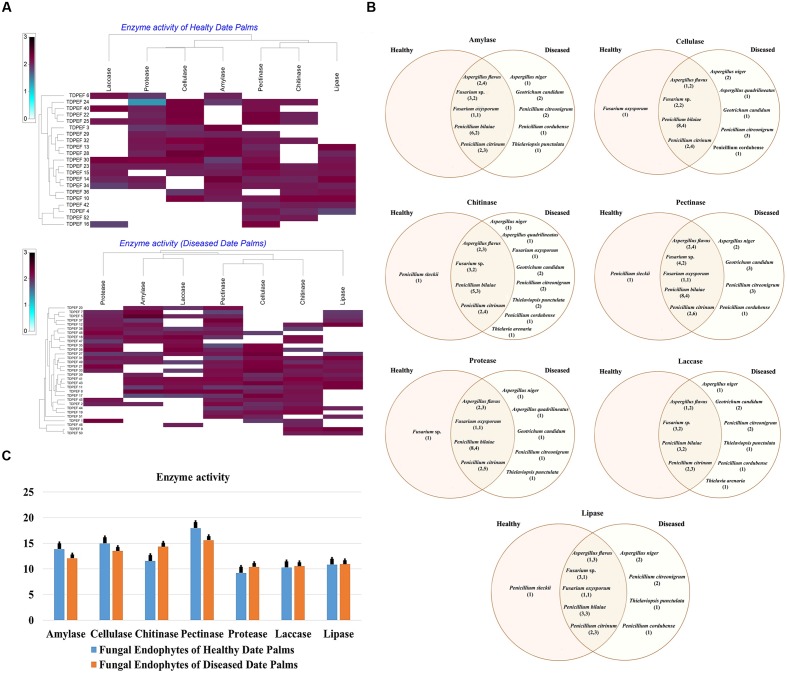
**(A)** Heat map of enzyme activity of fungal endophytes recovered from both of healthy and BLD date palm roots. **(B)** Venn diagram showing the distribution of endophytic fungal isolates in different enzyme activities including amylase, cellulose, chitinase, pectinase, protease, laccase, and lipase (the first number between brackets represents the number of isolates recovered from healthy roots and the second number represents that recovered from BLD roots). **(C)** Enzyme activity of fungal endophytes isolated from both of healthy and BLD diseased. Data presents mean ± standard error.

All 52 isolates were evaluated for their enzymatic activity with plate clearing assay for amylase, cellulase, chitinase, pectinase, protease, laccase, and lipase production. The results are presented in **Figure 2**.

#### Amylase Activity

Amylase activity has been studied in either healthy or BLD date palm roots fungal isolates. The results revealed a relevant capacity of the examined fungi to secrete these enzymes (**Figure 2**). Most of amylase producing fungi belonged to *Penicillium* followed by *Aspergillus* and *Fusarium* genera (**Figures 2A,B**). Mean amylase activity of all isolates of healthy roots was not significantly different from mean amylase activity of BLD isolates (**Figure 2C**).

#### Cellulase Activity

**Figure 2A** shows that among the recovered endophytic fungi, only 34 isolates displayed cellulase activity (**Figures 2A,B**). *Penicillium* species were able to degrade CMC at high percentage followed by *Aspergillus* and *Fusarium*. Among *Geotrichum* species, only *G. candidum* TDPEF 19 exhibited cellulase activity with halo diameter between 15 and 25 mm. Mean cellulase activity of all isolates of healthy roots was not significantly different from mean cellulase activity of BLD isolates (**Figure 2C**).

#### Chitinase Activity

All the isolated genera were able to grow on colloidal chitin agar plates reflecting their ability to produce extracellular chitinase (**Figure 2A**). Among the examined fungi, 25 chitinase-producing isolates belonged to Eurotiomycetes including *Penicillium* and *Aspergillus* species followed by nine Sordariomycetes and two *Geotrichum* species (**Figure 2B**). Mean chitinase activity of all isolates of healthy roots was not significantly different from mean chitinase activity of BLD isolates (**Figure 2C**).

#### Pectinase Activity

The findings revealed that the highest percentage of the isolated endophytic fungi (84.61%) exhibited pectinase activity (**Figure 2A**). The ability to degrade pectin substrates were detected mainly in endophytic fungi belonging to *Penicillium* species within *Thielavia* and *Thielaviopsis* genera (**Figure 2B**). Almost all of the isolated fungi showed halos diameter between 15 and 25 mm. Mean pectinase activity of all isolates of healthy roots were not significantly different from mean pectinase activity of BLD isolates (**Figure 2C**).

#### Protease Activity

As shown in **Figure 2A**, proteolytic activity was present in 59.61% of isolated endophytic fungi. Among the analyzed strains, 31 fungi showed degradation halos of casein on solid medium reflecting their ability to produce extracellular proteases. *Penicillium. Aspergillus*, and *Fusarium* are among the genera able to degrade casein (**Figure 2B**). Mean protease activity of all isolates of healthy roots was not significantly different from mean protease activity of BLD isolates (**Figure 2C**).

#### Laccase Activity

Half of isolated endophytic fungi from healthy and BLD date palm roots showed positive results for laccase screening on solid medium (**Figure 2A**). As shown in **Figure 2B**, fungal species belonged to Eurotiomycetes class that showed ability to produce this type of enzyme. Mean laccase activity of all isolates of healthy roots were not significantly different from mean protease activity of BLD isolates (**Figure 2C**).

#### Lipase Activity

The lipolytic activity of the fungal isolates are presented in **Figure 2A**. After irradiation with UV light of plates, 28 endophytic fungal strains showed fluorescent halos around their mycelia reflecting their ability to produce extracellular lipase. The lipase producing fungi were detected in *Penicillium* and within *Geotrichum* and *Thielavia* species (**Figure 2B**). Mean lipase activity of all isolates of healthy roots was not significantly different from mean lipase activity of BLD isolates (**Figure 2C**).

### *In vitro* Antimicrobial (Antagonistic) Assay of Date Palm Endophytes

**FIGURE 3:**
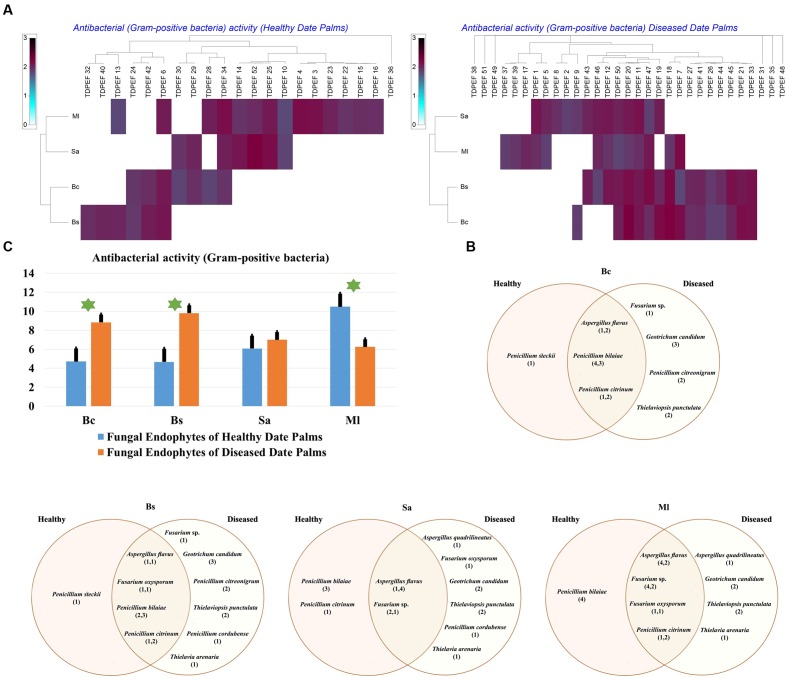
**(A)** Heat map of antibacterial (Gram-positive bacteria including *Bc. Bs. Sa*, and *Ml*) activity against fungal endophytes isolates from healthy and BLD date palm roots. **(B)** Venn diagram showing the distribution of endophytic fungal isolates in relation to different Gram-positive bacteria (the first number between brackets represents the number of isolates recovered from healthy roots and the second number represents the number of isolates recovered from of BLD roots). **(C)** Antibacterial (Gram-positive bacteria) activity against fungal endophytes isolates from healthy and BLD date palm roots. Data presents mean ± standard error. Bars labeled with asterisk are significantly different among the treatments at *P* < 0.05 using ANOVA analysis.

**FIGURE 4:**
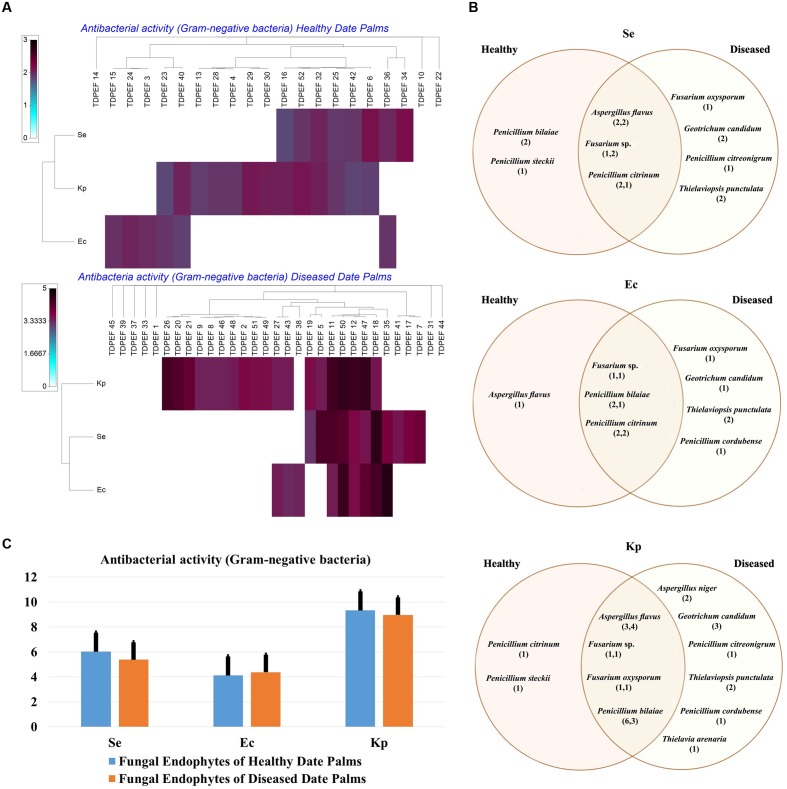
**(A)** Heat map of antibacterial (Gram-negative bacteria including *Se. Ec*, and *Kp*) activity against fungal endophytes isolates from healthy and BLD date palm roots. **(B)** Venn diagram showing the distribution of endophytic fungal isolates in relation to different Gram-negative bacteria (the first number between brackets represents the number of isolates recovered from healthy roots and the second number represents that recovered from BLD roots). **(C)** Antibacterial (Gram-negative bacteria) activity against fungal endophytes isolates from healthy and BLD date palm roots. Data presents mean ± standard error.

**FIGURE 5:**
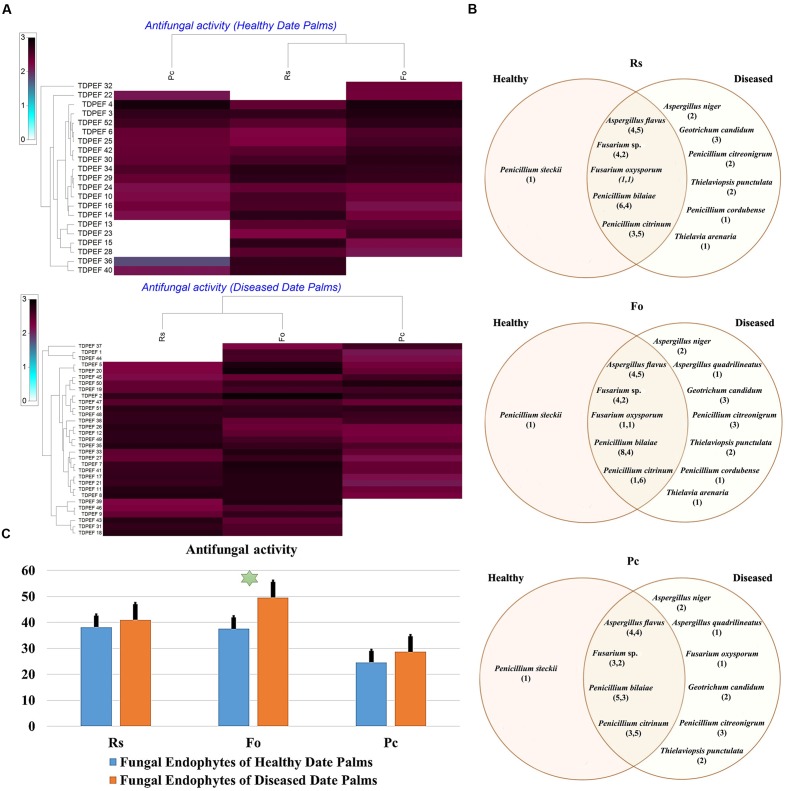
**(A)** Heat map of antifungal (fungal isolates including *Rs. Fo*, and *Pc*) activity against fungal endophytes isolates from healthy and BLD date palm roots. **(B)** Venn diagram showing the distribution of endophytic fungal isolates in relation to different fungal isolates (the first number between brackets represents the number of isolates recovered from healthy roots and the second number represents that recovered from BLD roots). **(C)** Antifungal activity against fungal endophytes isolates from healthy and BLD date palm roots. Data presents mean ± standard error. Bars labeled with asterisk are significantly different among the treatments at *P* < 0.05 using ANOVA analysis.

The isolated date palm healthy and BLD root endophytic fungi were examined against several pathogenic bacteria and phytopathogenic fungi to assess their antimicrobial activities. The results are presented in **Figures 3**–**5**. All isolates showed antimicrobial activity at least against two pathogenic microorganisms (**Figures 3**, **4**). For antibacterial activity, the average of the inhibition halo diameter was between 9.3 and 26.6 mm. The highest halo diameter was against *B. cereus* and *M. luteus* with *G. candidum* TDPEF 18 and *T. punctulata* TDPEF 47, respectively (**Figure 3A**). Only three endophytic fungi exhibited antibacterial activity against all tested bacteria including, *Fusarium* sp. TDPEF 11 and two *T. punctulata* species TDPEF 47 and TDPEF 50 (**Figures 3A,B**, **4A,B**). The Gram-negative bacteria *K. pneumoniae* was more sensitive than the other pathogenic bacteria. Among 52 isolates, 22 (42.30%) and 23 (44.23%) displayed antibacterial activity against both *B. cereus* and *B. subtilis*, respectively (**Figures 4A,B**). Furthermore, 20 (38.46%) and 27 (51.9%) endophytic fungi were able to inhibit the Gram-positive bacteria *S. aureus* and *M. luteus*, while 19 (36.53%) and 15 (28.84%) isolates could inhibit *S. enteritidis* and *E. coli*, respectively (**Figures 3A**, **4A**). Concerning antifungal activity, the percentage of inhibition was between 10.3 and 72 mm (**Figure 5A**). The best percentage of inhibition was obtained with *A. flavus* TDPEF 2 against *F. oxysporum*. The endophytic isolates were more active against pathogenic fungi than bacteria, as shown in **Figures 5A,B**. Only 18 isolates of date palm endophytes were unable to inhibit all the tested fungi. *F. oxysporum* was the most sensitive phytopathogenic fungus to date palm endophytic isolates. Among 52 endophytic fungi, 39 (75%) displayed antifungal activity against *P. catenulatum* (**Figure 5B**). A high number of isolates (85%) were able to inhibit the growth of both phytopathogenic fungi *F. oxysporum* and *R. solani* with wide spectrum activity (**Figures 5A,B**). Mean antibacterial activity against *B. cereus* and *B. subtilis* and antifungal activity against *F. oxysporum* of all isolates of healthy roots were significantly different from mean antibacterial activity of BLD isolates that have higher activities (**Figures 3C**, **5C**). Mean antibacterial activity against *S. aureus* and antifungal activity against *R. solani* and *P. catenulatum* show non-significant difference between healthy and BLD endophytic fungi, whereas, healthy date palm roots endophytes show significantly higher antimicrobial activities against *Ml* than BLD derived root endophytes (**Figures 3C**, **4C**, **5C**).

### Antimicrobial Assay of Date Palm Endophyte Extracts

**FIGURE 6:**
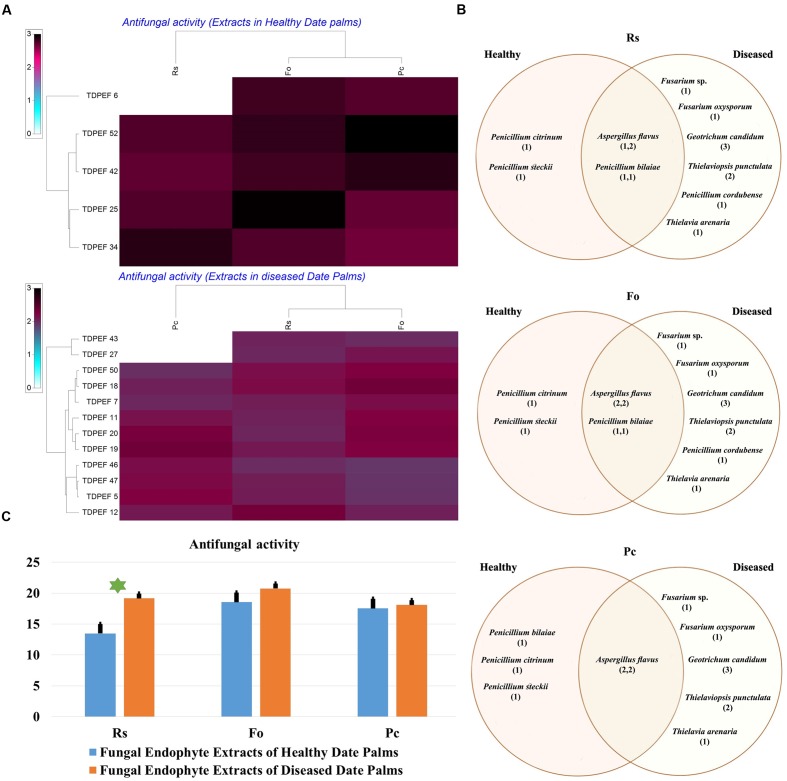
**(A)** Heat map of antifungal (fungal isolates including *Rs. Fo*, and *Pc*) activity against fungal endophytes extracts from healthy and BLD date palm roots. **(B)** Venn diagram showing the distribution of endophytic fungal isolates extracts in relation to different fungal isolates (the first number between brackets represents the number of isolates recovered from healthy roots and the second number represents that recovered from BLD roots). **(C)** Antifungal activity against fungal endophytes extracts from healthy and BLD date palm roots. Data presents mean ± standard error. Bars labeled with asterisk are significantly different among the treatments at *P* < 0.05 using ANOVA analysis.

**FIGURE 7:**
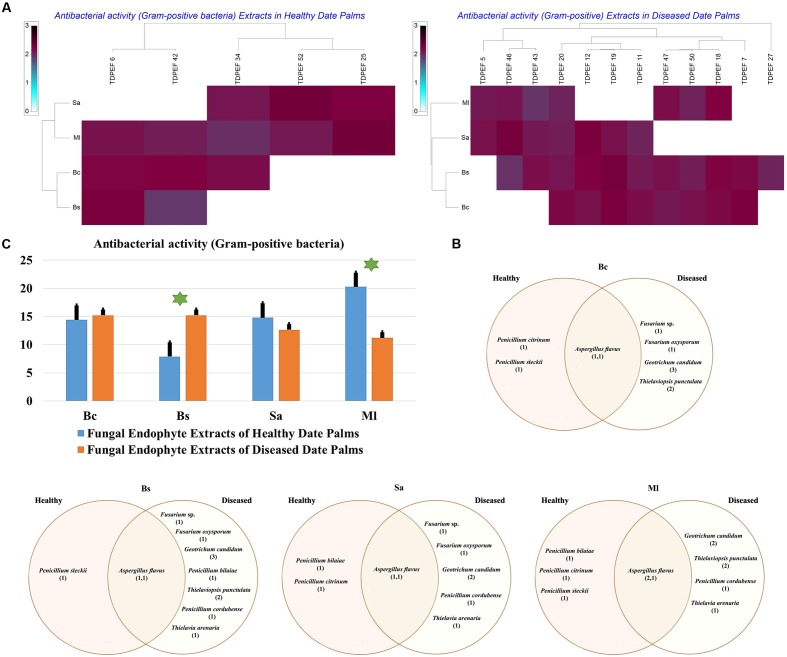
**(A)** Heat map of antibacterial (Gram-positive bacteria including *Bc. Bs. Sa*, and *Ml*) activity against fungal endophytes extracts from healthy and BLD date palm roots. **(B)** Venn diagram showing the distribution of endophytic fungal isolates extracts in relation to different Gram-positive bacteria (the first number between brackets represents the number of isolates recovered from healthy roots and the second number represents that recovered from BLD roots). **(C)** Antibacterial activity against fungal endophytes extracts from healthy and BLD date palm roots. Data presents mean ± standard error. Bars labeled with asterisk are significantly different among the treatments at *P* < 0.05 using ANOVA analysis.

**FIGURE 8:**
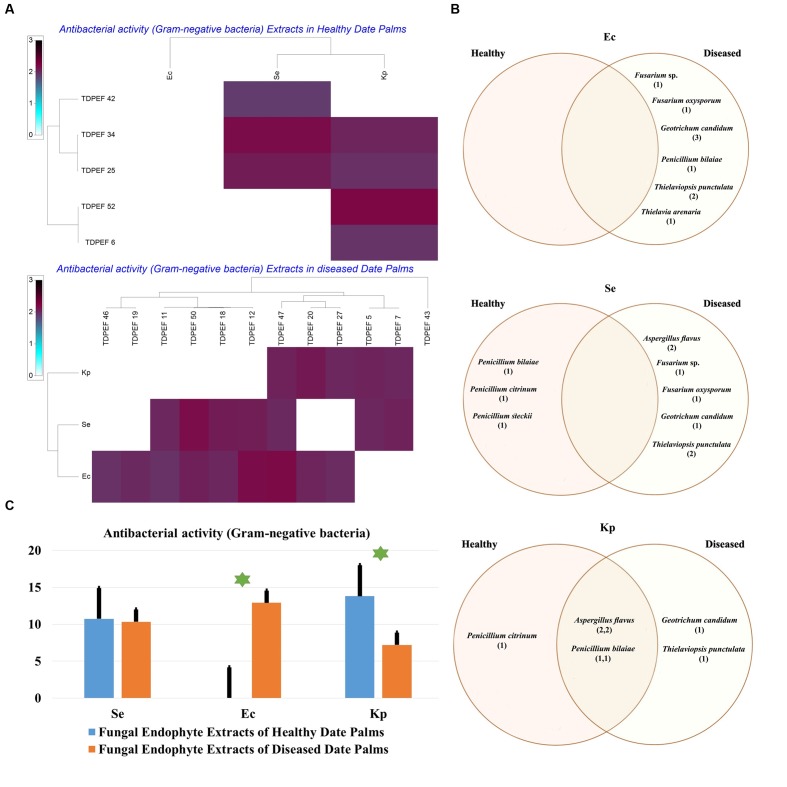
**(A)** Heat map of antibacterial (Gram-negative bacteria including *Se. Ec*, and *Kp*) activity against fungal endophytes extracts from healthy and BLD diseased date palm roots. **(B)** Venn diagram showing the distribution of endophytic fungal isolates extracts in relation to different Gram-negative bacteria (the first number between brackets represents the number of isolates recovered from healthy roots and the second number represents the number of isolates recovered from roots of BLD plant). **(C)** Antibacterial activity against fungal endophytes extracts from healthy and BLD date palm roots. Data presents mean ± standard error. Bars labeled with asterisk are significantly different among the treatments at *P* < 0.05 using ANOVA analysis.

The EtOAc extracts of 17 endophytic fungi were evaluated for their antibacterial and antifungal activity using agar-well diffusion method (**Figures 6**–**8**). Among the examined fungal extracts, only five originated from the isolates of healthy date palm roots. The 17 isolates submitted to fermentation assay were composed of four *Aspergillus*, two *Fusarium*, three *Geotrichum*, two *Thielaviopsis*, five *Penicillium* species, and *T. arenaria* TDPEF46. The inhibitory activity of fungal extracts expressed in term of diameter of inhibition zones were presented in **Figures 6A**, **7A**, **8A**. The halo inhibition diameter ranged from 12.6 to 28.6 mm for antibacterial activity, while for antifungal activity the inhibition diameter was between 13.6 and 30 mm. Among the 17 fungal extracts, 11 showed promising growth inhibitory activity against *B. cereus*. A high number of EtOAc extracts were able to inhibit the growth of *B. subtilis* with halo diameter ranging from 14.3 to 28 mm (**Figure 6B**). Nine extracts of date palm endophytic fungi exhibited significant inhibitory activity against *S. enteritidis. E. coli*, and *K. pneumoniae* (**Figure 7B**). Furthermore, all the examined extracts displayed antagonistic activity against *F. oxysporum* (**Figure 8B**). The EtOAc extracts of *G. candidum* and the two strains of *T. punctulata* exhibited the highest levels of antagonistic activity thanks to their ability to inhibit the growth of six test bacteria and three phytopathogenic fungi. Concerning antibacterial activity, the highest halo diameter was recorded with the extract of *P. bilaiae* TDPEF 25 against the Gram-positive bacteria *M. luteus* (**Figure 6B**). In addition, the EtOAc extract of *G. candidum* TDPEF 19 displayed the highest level of antifungal activity with halo diameter of 30 mm against *P. catenulatum* (**Figure 8B**). Mean antibacterial activity against *Bs* and *Ec* and antifungal activity against *Rs* of all isolates of healthy roots were significantly different from mean lipase activity of BLD isolates that have higher activities (**Figures 6C**, **7C**, **8C**). Mean antibacterial activity against *Bc. Sa*, and *Se* and antifungal activity against *Fo* and *Pc* show non-significant difference between healthy and BLD endophytic fungi, whereas, healthy date palm roots endophytes show significantly higher antimicrobial activities against *Ml* and *Kp* than BLD derived root endophytes (**Figures 6C**, **7C**).

### Date Palm Endophytes versus Endophyte Extracts Antimicrobial Activities

**FIGURE 9:**
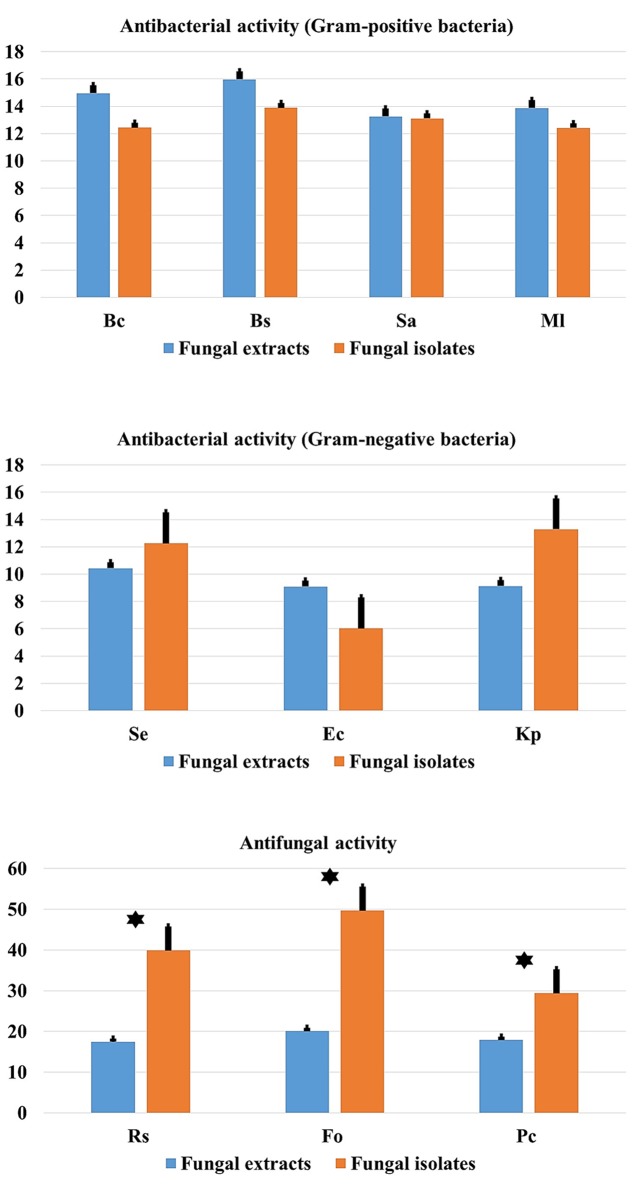
**Comparison of antibacterial (Gram-positive and Gram-negative) and antifungal activity in relation to endophytic fungal isolates and extracts.** Data presents mean ± standard error. Bars labeled with asterisk are significantly different among the treatments at *P* < 0.05 using ANOVA analysis.

Comparison between mean antimicrobial activity against Gram-positive and Gram-negative bacteria and phytopathogenic fungi and antimicrobial activity of fungal endophytes extracts are reported in **Figure 9**. Results clearly suggest that endophytic root fungal extracts account for all the observed antimicrobial activity when using endophytes themselves. However, extracts from endophytes significantly account approximately for half of the antimicrobial activity obtained when using endophytes *in vitro* assays against fungal phytopathogens (*p* < 0.01).

### Determination of MIC, MBC, and MFC of Endophytic Fungal Extracts

The EtOAc extract of the endophytic fungi *G. candidum* TDPEF20 and *T. punctulata* TDPEF47 were subjected to micro- dilution plate in order to determine their MIC, MBC, and MFC values. As shown in **Table 1**, the EtOAc extract of *G. candidum* was more active against *Bc. Ml*, and *Rs* (MIC of 0.56 μg/μL), followed by *Bs. Ec*, and *Fo*. In addition, *Geotrichum* extract exhibited both bacteriostatic and fungistatic effects (MBC/MIC and MFC/MIC ≥4). Concerning *Thielaviopsis* extract, its MIC values ranged from 0.78 to 6.25 μg/μL. However, *Thielaviopsis* extract exhibited the best inhibitory activity against *Bs* and *Kp*. The recorded MBC values indicated the bacteriostatic actions of *Thielaviopsis* extract. It could predominantly inhibit completely the fungal growth (MFC/MIC ≥4) in exception of *Pc.* The results obtained in terms of MIC, MBC, and MFC indicated that *G. candidum* extract displayed a higher bactericidal and fungicidal effect than *T. punctulata* extract.

**Table 1:** Minimum inhibitory, minimum bactericidal and minimum fungicidal concentrations of extracts of *G. candidum* TDPEF 20 and *T. punctulata* TDPEF 47 against human bacterial pathogens and fungal phytopathogens.

	TDPEF 20		TDPEF 47	
		
	Concentration (μ g/μ L)			
	
	MIC	MBC	MIC	MBC
**Gram-positive bacteria**				
*Bacillus subtilis*	1.56	6.25	0.78	6.25
*Bacillus cereus*	0.78	12.5	1.56	12.5
*Staphylococcus aureus*	3.12	12.5	-	-
*Micrococcus luteus*	0.78	6.25	1.56	6.25
**Gram-negative bacteria**				
*Salmonella enteric serotype enteritidis*	-	-	6.25	50
*Escherichia coli*	1.56	6.25	3.12	25
*Klebsiella pneumoniae*	6.25	25	3.12	12.5

**Phytopathogen fungi**	**MIC**	**MFC**	**MIC**	**MFC**

*Rhizoctonia solani*	0.78	6.25	3.12	12.5
*Fusarium oxysporum*	1.56	6.25	6.25	25
*Pythium catenulatum*	3.12	25	3.12	6.25


## Discussion

Endophytes especially fungi produce an impressive myriad of bioactive molecules and enzymes that cope with pathogen infection of plants (Newman and Cragg, 2016; Vasundhara et al., 2016). Natural compounds produced by endophytic fungi have been shown to interfere with key host and pathogen processes required for successful infection of the host and to mitigate the adverse effects of broad variety of animal and plant pathogens (Stierle and Stierle, 2015; Mousa et al., 2016; Tanney et al., 2016). Endophytic fungi have tremendous impacts on their host plants inducing their tolerance to biotic and abiotic stresses, promoting their growth and the production of wide variety of secondary metabolites (Jia et al., 2016). Therefore, it is believed that some bioactive compounds produced by some medicinal plants are actually the result of the secondary metabolite biosynthesis by their endophytes. This is the case of the highly oxygenated diterpenoid natural product Taxol, one of the most widely used anticancer drugs, first isolated from the pacific yew tree (*Taxus brevifolia*) and then attributed to its endophyte *Taxomyces andreanae* (Stierle et al., 1993).

Geographic locality, host identity, developmental stage, and biotic and abiotic stresses are considered the main drivers of plant fungal endophyte diversity and believed to shape their communities. While fungal endophyte characterization in healthy tissues have been extensively studied, few reports addressed these communities in plants subjected to abiotic or biotic stresses (Douanla-Meli et al., 2013). In the current study, endophytic fungi from healthy and BLD roots of date palm trees widely cultivated in many tropical and subtropical regions worldwide were studied for their phylogenetic affinities and bioactive potential. Date palm tree was favored because of its wide use in folk medicine mainly for its antioxidant, wound healing and antimicrobial activities (Abdennabi et al., 2016). Furthermore, extracts of different parts of date palm trees including fruits and pollen showed potential inhibitory activity against several pathogens (Saleh and Otaibi, 2013; Daoud et al., 2015). In addition, Siala et al. (2016) reported the potential of endophytic bacteria isolated from healthy date palm (*P. dactylifera* L.) against phytopathogenic fungi namely *F. oxysporum* f. sp. *albedinis*, the causal agent of bayoud disease. Date palms have the ability to survive in arid regions for several years, therefore, their endophytic community may produce wide range of bioactive components during various stages of their life cycle. Roots of date palm were chosen for isolation of endophytic fungi owing to their significant content of endophytic communities (Wearn et al., 2012). In addition, previous researches have revealed that most of endophytes isolated from foliar tissues were already known to occur commonly in the roots of the host plants (Moricca et al., 2012). A total of 52 endophytic fungi was isolated from roots of date palm (*P. dactylifera* L.). These isolates were identified on the basis of their 18S rDNA sequences analysis and shown to represent 13 species belonging to five genera. Some of isolates (36.8%) were shared between healthy and diseased root fungal microbiomes, whereas 18.4 and 44.7% of the isolates were specific to healthy and BLD root fungal microbiomes, respectively (**Figures 1A–C**). Shared species found to belong to genera *Penicillium. Fusarium*, and *Aspergillus. Penicillium stecki* was specific to healthy tissues, whereas *A. niger. A. quadrilineatus. P. citreonigrum. P. cordubense. G. candidum. T. punctulata*, and *T. arenaria* were specific to BLD root fungal microbiome (**Figure 1C**).

Our results disagree with those of Ben Chobba et al. (2013) who recorded the dominance of *Alternaria. Pythium*, and *Curvularia* genera in roots of date palm belonging to the same variety Deglet Ennour. However, many factors such as time of sampling, age of host plant, soil conditions as well as dynamics of soil mycobiota may have influence on endophytes of date palm trees and can explain therefore this discrepancy. Variations in fungal endophytes and their frequency of isolation have been reported for many host plants (Collado et al., 2001; Nalini et al., 2014). Most of our isolates have been reported as endophytes in other plants, including mangrove, *Catharanthus roseus. Moringa oleifera. Paspalum maritimum, Pinus thunbergii*, and *Coffee arabica* (Vega et al., 2006; Correa et al., 2011; Elavarasi et al., 2012; Kumar et al., 2013; Rajeswari et al., 2016). Moreover, *Fusarium* and *Aspergillus* genera were also previously isolated as endophytic fungi from the plants of *Opuntia dillenii* that survive in arid regions (Ratnaweera et al., 2015).

One of the most abundant genera in the roots of date palm was *Penicillium*, which have been isolated as endophyte from different photosynthetic plants such as *Nicotiana* spp., *Pasania edulis* Makino and *Vigna radiata* L. Recent research excluded the pathogenicity of *Penicillium* species in symbiotic lifestyle with plant tissues (Diaz et al., 2011). In our study, only two species of *Penicillium* were isolated from either healthy and/or BLD roots of date palm trees, including *P. bilaiae* and *P. citrinum*. These two species are ubiquitous saprobes that promote seedling and plant growth (Mushtaq et al., 2012). However, *G. candidum* is known as pathogenic fungus of citrus, tomato, cucumber, grapefruit, and carrot causing sour rot disease (Suprapta et al., 1995). We believe that the isolation of *G. candidum* in the oasis of Nefta might be related to the cooled system of geothermal water used for irrigation of greenhouse cultures (Ben Mohamed and Said, 2008). The project of greenhouses was recently established in south regions of Tunisia and the crops produced were composed of tomatoes, cucumber, melons and watermelons. Among the endophytic fungi from BLD roots of date palm, *T. punctulata* was isolated for the first time in North Africa and never described to date as endophyte. This fungus, previously known as *Ceratocystis radicicola*, is the responsible agent of several date palm disorders called black scorched leaves (Al-Naemi et al., 2014). Recent reports revealed that *T. punctulata* occur especially on stressed date palm trees in areas where salinity and drought are preponderating (Zaid et al., 2002). Greenhouse cultures around Nefta oasis are irrigated using a slightly saline water, which could provide additional clues to explain the presence of *T. punctulata*. We therefore recommend strict control of salinity of irrigation water in the oasis in order to avoid stress of date palm trees.

Any speculation about putative involvement of *G. candidum* or *T. punctulata* in the development of BLD in date palm trees needs additional investigations. Several studies have shown the detection and isolation of pathogenic fungi in endophytic communities of diverse host plants (Sun et al., 2012; Wearn et al., 2012). This confirm the observation of Pawłowska et al. (2014) which suggested that endophytism is a period in the life cycle of pathogenic microorganisms (Schulz and Boyle, 2005).

Recently, the endophytes isolated from arid zones are becoming increasingly recognized as potential source for new enzymes and secondary metabolites. Our findings revealed that endophytic fungi of date palm are able to produce a broad range of valuable enzymes including amylase, cellulase, chitinase, pectinase, protease, laccase, and lipase. In the present study, all endophytic fungi were able to produce at least two of the seven evaluated enzymes, attesting the metabolic diversity within date palm endophytes (**Figures 2A,B**). Jrad et al. (2014) reported the potentiality of both healthy and BLD date palm bacteria to produce diverse hydrolytic enzymes. In line with our findings *Fusarium. Penicillium*, and *Aspergillus* genera have been reported to produce an extensive range of extracellular enzymes (Kwon et al., 2007; Alp and Arikan, 2008; Park et al., 2016). Additionally, almost all of the assayed enzymes in the current study were known to be produced by endophytic microorganisms and are needed for both colonization of host plant and decomposition of dead tissues (Saikkonen et al., 2004). There was no statistical support for a higher mean activity of the different enzymes in BLD endophytes compared to healthy roots fungal endophytes (**Figure 2C**).

All endophytic fungi recovered in this study were screened for their antibacterial and antifungal potential on solid media. Preliminary screening allows the detection of the microorganisms that possess interesting antimicrobial activity. Almost all isolates exhibited antimicrobial activity against at least two of the test microorganisms, including 7 Gram-positive and Gram-negative bacteria and three fungi, on solid media (**Figures 3A,B**, **4A,B**, **5A,B**). In total, 17 (32.69%) isolates among endophytic fungi exhibited potential antimicrobial activity and were further subjected to fermentation assay. This percentage is comparable to the percentage recovered in the study of Santos et al. (2015) using *Indigofera suffruticosa* Miller (33.6%). This result highlight the enormous capacity of bioactive molecules production of these endophytes as suggested by Santos et al. (2015). Comparing mean antimicrobial activities between healthy and BLD root endophytes revealed that no clear tendency suggests superior activity of one group to the others. Therefore, we can conclude that using stressed roots for recovery of isolates offers new different endophytes than normal roots but does not provide isolates with higher antimicrobial activities (**Figures 3C**, **4C**, **5C**).

The 17 most active isolates have been selected and were submitted to fermentation in liquid medium, extraction of bioactive secondary metabolites using EtOAc and subsequent test of their antimicrobial activities using agar diffusion method. EtOAc was chosen for its ability to extract both polar and non-polar bioactive metabolites present in the fungal mycelia. All extracts were very efficient in inhibiting both Gram-positive and Gram-negative bacteria and fungi (**Figures 6A,B**, **7A,B**, **8A,B**). Interestingly, these fungal endophyte extracts proved effective against the three fungal pathogens used. Comparing mean antimicrobial activities between healthy and BLD root endophytes extracts revealed also that no clear tendency suggests superior activity of one group to the other (**Figures 6C**, **7C**, **8C**).

Comparison between mean antimicrobial activity against Gram-positive and Gram-negative bacteria and phytopathogenic fungi and antimicrobial activity of fungal endophytes extracts, (**Figure 9**) clearly suggest that endophytic fungal extracts responsible for all the observed antimicrobial activity when using endophytes themselves. However, extracts from endophytes significantly account for approximately half of the antimicrobial activity obtained when using endophytes as *in vitro* assays against fungal phytopathogens (*p* < 0.01). This finding suggests that mechanisms other than the production of secondary metabolites account for the remaining 50% of the antifungal activity observed by living endophytes. We speculate that this could be related to the presence of alternative biocontrol strategies such as the production of chitinases for example that are known to inhibit the growth of fungal phytopathogens (Da Silva et al., 2016). However, more detailed research work is required to confirm this hypothesis.

The two most active isolates *G. candidum* and *T. punctulata* were submitted to the microdilution method to evaluate precisely their MIC, MBC, and MFC. Extract of *G. candidum* was more active against *B. cereus. M. luteus*, and *R. solani* (MIC of 0.56 μg/μL). *Thielaviopsis* extract exhibited the best inhibitory activity against *B. subtilis* and *K. pneumoniae*. In line with our findings, endophytes from *Geotrichum* genus have been reported as source of nematicidal, antituberculosis, antifungal, and antimalarial compounds (Kongsaeree et al., 2003; Li et al., 2007). Most of the compounds isolated from the endophytes of *Geotrichum* sp. are almost isocumarin and triterpenoids. Production of such compounds by endophytic fungi has been reported by recent review (Mousa and Raizada, 2013). No previous researches have isolated *Thielaviopsis* as an endophyte which warrants serious investigation to study its full antimicrobial potential. Our results indicate that endophytic fungi of healthy and BLD roots of date palm constitute a potent source of useful antibacterial and antifungal compounds. The next step will be to establish a strain collection bank with high throughput antibacterial and antifungal screening in addition to performing large scale fermentation of the potential microbial hits to identify the bioactive metabolites responsible for such activities in these extracts.

## Author Contributions

Conceived and designed the experiments: FM, AD, LB, and NG. Performed the experiments: FM, LB, AD, ACB, LL, FA, and MR. Analyzed the data: FM, LB, AD, ACB, LL, MR, and NG. Contributed reagents/materials/analysis tools: LB, LL, FA, and NG. Wrote and enriched the literature: LB, FM, AD, ACB, LL, FA, AK, MR, and NG.

## Conflict of Interest Statement

The authors declare that the research was conducted in the absence of any commercial or financial relationships that could be construed as a potential conflict of interest.
